# Deuterium-labeled Raman tracking of glucose accumulation and protein metabolic dynamics in *Aspergillus nidulans* hyphal tips

**DOI:** 10.1038/s41598-020-80270-9

**Published:** 2021-01-14

**Authors:** Mitsuru Yasuda, Norio Takeshita, Shinsuke Shigeto

**Affiliations:** 1 Department of Chemistry, School of Science and Technology, Kwansei Gakuin University, Sanda, Hyogo 669-1337 Japan; 2 Microbiology Research Center for Sustainability (MiCS), Faculty of Life and Environmental Sciences, University of Tsukuba, Ibaraki, Tsukuba 305-8572 Japan; 3 Present Address: Department of Pharmacology, School of Medicine, Keio University, Tokyo, 160-8582 Japan

**Keywords:** Fungi, Physical chemistry, Imaging

## Abstract

Filamentous fungi grow exclusively at their tips, where many growth-related fungal processes, such as enzyme secretion and invasion into host cells, take place. Hyphal tips are also a site of active metabolism. Understanding metabolic dynamics within the tip region is therefore important for biotechnology and medicine as well as for microbiology and ecology. However, methods that can track metabolic dynamics with sufficient spatial resolution and in a nondestructive manner are highly limited. Here we present time-lapse Raman imaging using a deuterium (D) tracer to study spatiotemporally varying metabolic activity within the hyphal tip of *Aspergillus nidulans*. By analyzing the carbon–deuterium (C–D) stretching Raman band with spectral deconvolution, we visualize glucose accumulation along the inner edge of the hyphal tip and synthesis of new proteins from the taken-up D-labeled glucose specifically at the central part of the apical region. Our results show that deuterium-labeled Raman imaging offers a broadly applicable platform for the study of metabolic dynamics in filamentous fungi and other relevant microorganisms in vivo.

## Introduction

Hyphal tips are a unique site where polarized growth of filamentous fungi occurs. By elongating their hyphae at the tips, fungi invade plant and animal host cells as pathogens, often causing fungal infections^1,2^; on the other hand, they exhibit a high capability to secrete diverse (exo)enzymes^3,4^. Such multifaceted functions of filamentous fungi and their significance in the medical, bioindustrial, and agricultural fields have stimulated intensive studies of the molecular mechanism for hyphal tip growth^5–7^. Because continued growth at the hyphal tip requires metabolic energy, it is of vital importance for full elucidation of the growth mechanism to visualize metabolic activity in the hyphal tip cell. Nonetheless, most of the currently available techniques for metabolic imaging of cells and tissues, such as NMR^8^ and positron emission tomography^9^, have insufficient spatial resolution. Nanoscale secondary ion mass spectrometry coupled with stable isotope probing (SIP) can measure the distributions of stable isotopes with high spatial resolution of < 50 nm and has been exploited in biology^10,11^, but it is destructive in nature. Thus, tracking dynamic changes in metabolic state and its spatial heterogeneity in vivo remains a big challenge.

Raman microspectroscopy, being able to look at molecular vibrations in any form of microscopic samples in a nondestructive manner, provides a promising tool for in vivo metabolic imaging when coupled with the SIP method. In addition to the nondestructive character, it has high chemical specificity and (diffraction-limited) subcellular spatial resolution^12^, both of which are necessary for probing different metabolisms in different intracellular regions. SIP bestows on Raman microspectroscopy the ability to probe the enrichment of stable isotopes (e.g., ^2^H and ^13^C) in chemical bonds. This is because substitution with heavier isotopes leads to a decrease in the frequencies of vibrational modes that are associated with the bonds. Whether the magnitude of the isotopic down-shift is large, moderate, or practically undetectable depends considerably on vibrational modes (stretching, bending, deformation, etc.). The intensities of those isotopically shifted bands are a quantitative measure of the degree to which stable isotopes are enriched.

In applications to microorganisms, Huang and coworkers used deuterium- and ^13^C-labeled glucose or heavy water (D_2_O) as a stable-isotope tracer to identify functionally different single cells within microbial communities^13–18^. Noothalapati and Shigeto applied Raman imaging with ^13^C-labeled glucose to reveal colocalization of newly synthesized proteins to lipid droplets in the fission yeast (single-celled fungus) *Schizosaccharomyces pombe*^19^. They also developed a combination of ^12^C/^13^C-mixed labeling strategy and multivariate curve resolution–alternating least squares^20–22^ (MCR–ALS) and studied ergosterol biosynthesis in single living *S. pombe* cells^23^. Following these studies, the Min group recently implemented the SIP method into stimulated Raman scattering (SRS) microscopy^24,25^, a nonlinear variant of Raman spectroscopy, for imaging metabolic dynamics in animal cells and tissues^26,27^. The multicellular microorganism, filamentous fungi, represents an interesting group of cells that have not yet been explored by the SIP-based Raman approaches.

Here, with the ultimate goal of revealing the relationship between metabolic activity and hyphal tip growth, we leveraged deuterium-labeled Raman imaging to track in vivo spatiotemporally heterogeneous glucose accumulation and assimilation into proteins in hyphal tips of the model filamentous fungus *Aspergillus nidulans*. Several molecular mechanisms of hyphal tip growth have been revealed in *A. nidulans*^7^, such as polarity maintenance^6^, cell cytoskeletons^5^, membrane transport^7,28^, and spatial coupling of apical exocytosis and subapical endocytosis^29,30^, which is of particular importance here. Previously, we obtained static information on the distributions of multiple biomolecular components in the tip, branching, and basal regions of *A. nidulans* hyphae by using MCR–ALS-assisted Raman microspectroscopy^31^. We now focus on the visualization of dynamic metabolic activities at the site of fungal growth. We measured extensive Raman spectra of *A. nidulans* hyphal tip cells containing the carbon–deuterium (C–D) stretching band as well as other D-substituted and unsubstituted bands of proteins, at different times after the fungus was supplied with D-labeled glucose. The time-lapse Raman images obtained by a band deconvolution analysis showed that the glucose taken up by the hypha is accumulated along the inner edge of the tip cell. We also found that the Raman signal of D-labeled newly synthesized proteins increases approximately 1.8 times faster in the central part of the apical region (~ 5 μm from the tip apex) than in the rear region, an important finding strongly suggestive of active protein synthesis and the subsequent transport of newly synthesized proteins. Our results demonstrate a highly heterogeneous nature of metabolic activity in the apical region of *A. nidulans* hyphae.

## Results

### Temporal changes in averaged Raman spectra

**Figure 1:**
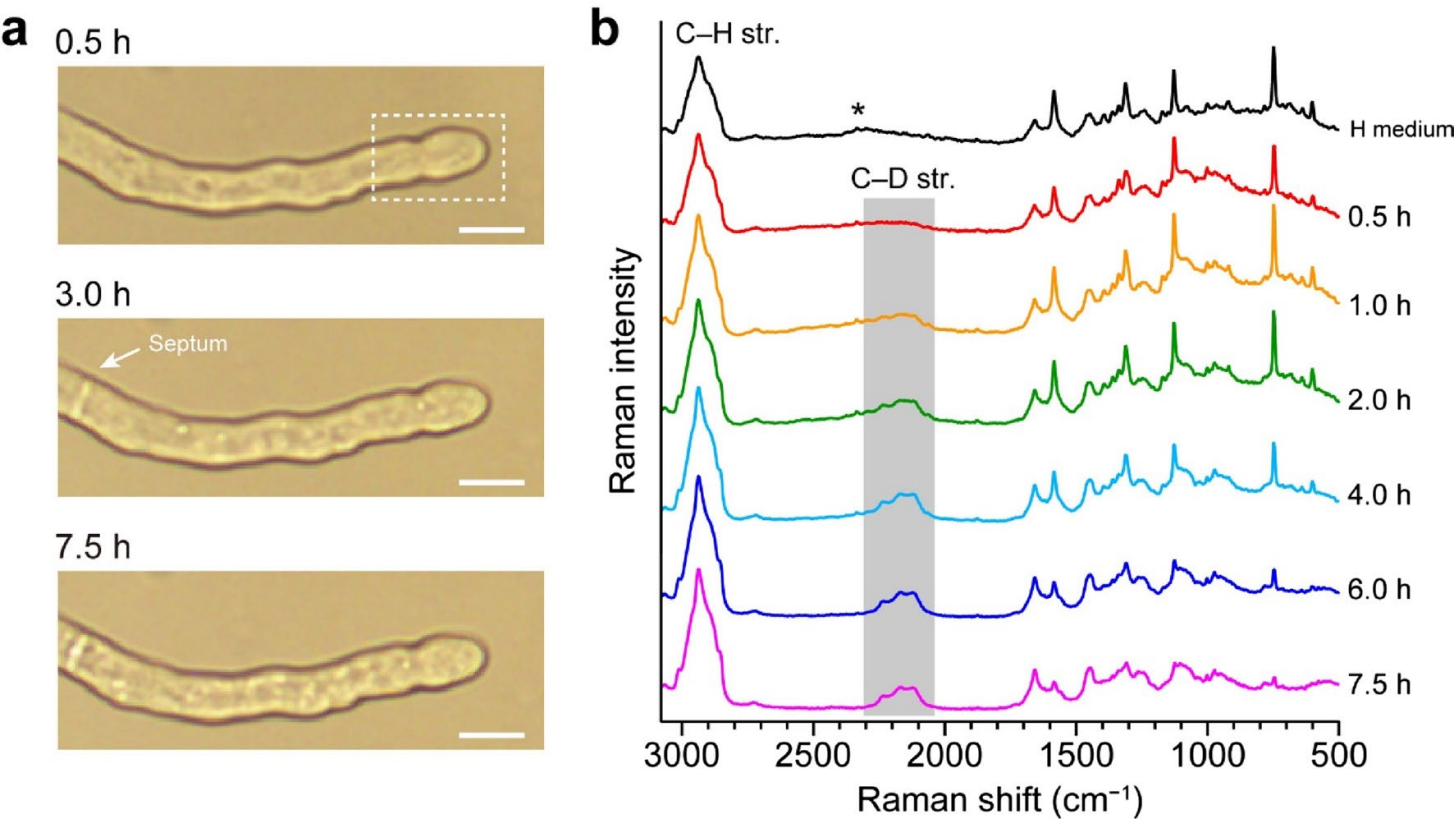
Averaged Raman spectra of an *A. nidulans* hyphal tip at different culture times in D medium. (**a**) Optical images at 0.5, 3.0, and 7.5 h of the *A. nidulans* hypha studied in the present work. The formation of a septum is seen after 3.0 h. White dashed rectangular in the top image indicates the scanned area. Scale bar = 5 μm. (**b**) Average of Raman spectra measured within the hypha at 0.5, 1.0, 2.0, 4.0, 6.0, and 7.5 h after replacing H medium with D medium, together with a typical Raman spectrum of *A. nidulans* hyphal tips in H medium. The spectra are vertically offset for clarity of display. Asterisk denotes a broad background due to fluorescence emission from reduced cytochrome *c*^36^. str. = stretching.

The *A. nidulans* hyphal cell we studied in this work is shown in Fig. 1a. The cell culture and Raman imaging apparatus are described in detail in the Methods section. We performed Raman imaging of the apical region within ~ 10 μm from the apex of the hypha (see the dashed rectangular in the top image of Fig. 1a). It is seen from the optical images that this hypha was not growing at its tip. This lack of growth may raise a concern about the physiological state of the hypha, but the formation of a septum at ~ 3.0 h suggests that the tip cell was at least alive. We will elaborate on the halted growth in the Discussion section.

To identify Raman signatures that are suitable for probing specific D-substituted biomolecules, we first examined a time course of averaged Raman spectra (Fig. 1b). Each spectrum is an average of all Raman spectra recorded inside the hypha. The spectrum acquired after only 0.5 h of incubation in D medium (Fig. 1b, red line) is almost identical to a typical Raman spectrum of *A. nidulans* hyphal tips grown in H medium (Fig. 1b, black line). Here and hereafter, culture media supplemented with unlabeled and D-labeled glucose are referred to as H and D media, respectively. The 0.5 h spectrum consists of three distinct spectral windows: the intense, broad C–H stretching Raman band (2800–3000 cm^−1^); a “silent region” with no prominent peaks (1800–2700 cm^−1^); and the so-called fingerprint region where there are many sharp Raman bands (500–1800 cm^−1^). In our previous work, we have already characterized the prominent bands observed in the typical *A. nidulans* Raman spectrum^31^, and their assignments can be found in Supplementary Table S1. The noticeable Raman bands at 601, 748, 1128, 1313, and 1583 cm^−1^ in the fingerprint region are all assigned to the vibrational modes of the porphyrin ring of cytochrome *c*^32^, which are resonance-enhanced with 532-nm excitation^31,33–35^. These cytochrome bands can be used as a marker band for mitochondria.

As the culture time in D medium increases, a new band peaking at ~ 2150 cm^−1^ emerges in the silent spectral window. This band is unambiguously assigned to the C–D stretching band^15,18,26,27^, which is a clear manifestation of the isotopic shift of the C–H stretching band and the enrichment of the C–D bond in the hypha. It becomes discernible at 1.0 h and grows in intensity within a few hours. Although this C–D stretching band is useful for easily detecting the D incorporation, it is not possible to determine what biomolecular species and how much of them are substituted with deuterium just by looking at the intensity of the entire band because it represents the gross amount of any C–D bond-containing biomolecules. We resolved this problem of poor biomolecular specificity by investigating the shape of the C–D stretching band.

### Identification of glucose- and protein-dominating C–D stretching bands

**Figure 2:**
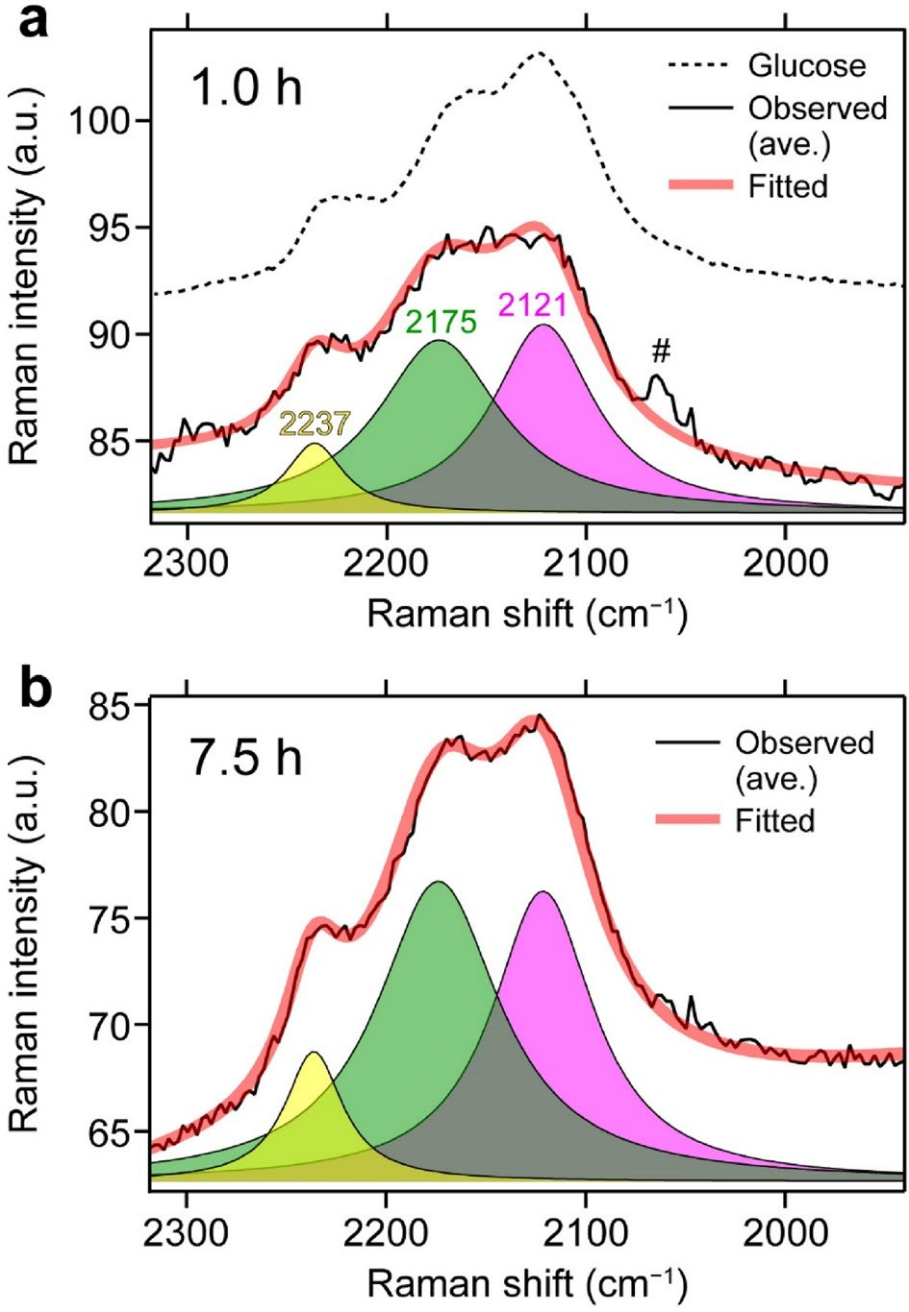
Least-squares fitting analysis of the C–D stretching band assuming three Lorentzian functions and a linear baseline. Observed averaged (thin black line) and fitted (thick red line) spectra at 1.0 (**a**) and 7.5 (**b**) h, together with the deconvolved subbands at 2121 (magenta), 2175 (green), and 2237 (yellow) cm^−1^. Also displayed in **a** is the Raman spectrum of D-labeled glucose dissolved in water (dashed line; not to scale). Hash sign (#) in **a** denotes a combination band of cytochromes.

A detailed scrutiny of the Raman imaging data showed observable, albeit small, changes in the C–D stretching band shape that are position- and time-dependent, so we undertook least-squares curve fitting to deconvolve the band. As can be seen from Fig. 2, the C–D stretching band appears to comprise at least three subbands. Thus, we analyzed the C–D stretching band (1940–2318 cm^−1^) in each space-resolved Raman spectrum using three Lorentzian functions and a linear function that accounts for an inclined baseline. We first fit the averaged C–D stretching band profile and determined the peak position and band width of the three Lorentzian subbands. The band parameters so determined are summarized in Supplementary Table S2. A peak at ~ 2062 cm^−1^ (marked with hash sign in Fig. 2a), which is a combination band of cytochromes^36,37^, was so weak that it was excluded from the fitting without much deterioration of fit quality. Examples of the fitted results shown in Fig. 2a (1.0 h) and b (7.5 h) indicate the goodness of the fitting. Furthermore, they illustrate that the relative height of the 2121 and 2175 cm^−1^ bands does differ between 1.0 and 7.5 h. We then fit the individual spectra while keeping the peak position and band width fixed throughout the fitting; that is, the only adjustable parameters were the height (peak intensity) of each Lorentzian subband and the slope and intercept of the linear baseline.

Our next task was to associate each of the three subbands with a specific biomolecular component. Plausible candidates include D-labeled glucose taken up by the hyphal cell, and proteins, DNAs, and lipids newly produced from anabolic metabolism of D-labeled glucose. DNAs and lipids can safely be ruled out, because nucleic acids typically show much lower Raman intensities than proteins and because the hyphal tip region is not very abundant in lipids according to our previous study^31^. We attribute the 2121 cm^−1^ band predominantly to D-labeled glucose and the 2175 cm^−1^ band to D-labeled proteins. The rationale for this assignment is as follows: (1) The 2121 cm^−1^ band has a peak close to that of the C–D stretching band of D-labeled glucose dissolved in water (Fig. 2a, dashed line). (2) The time-lapse Raman image of the 2121 cm^−1^ band exhibits a localized distribution along the inner edge of the hyphal tip that reflects accumulation of D-labeled glucose taken into the cell (see below). (3) Previous studies showed that D-labeled proteins isolated from animal tissues show a C–D stretching band at around 2190 cm^−1^ (Ref.^26,27^), which is in reasonable agreement with our 2175 cm^−1^ band.

**Figure 3:**
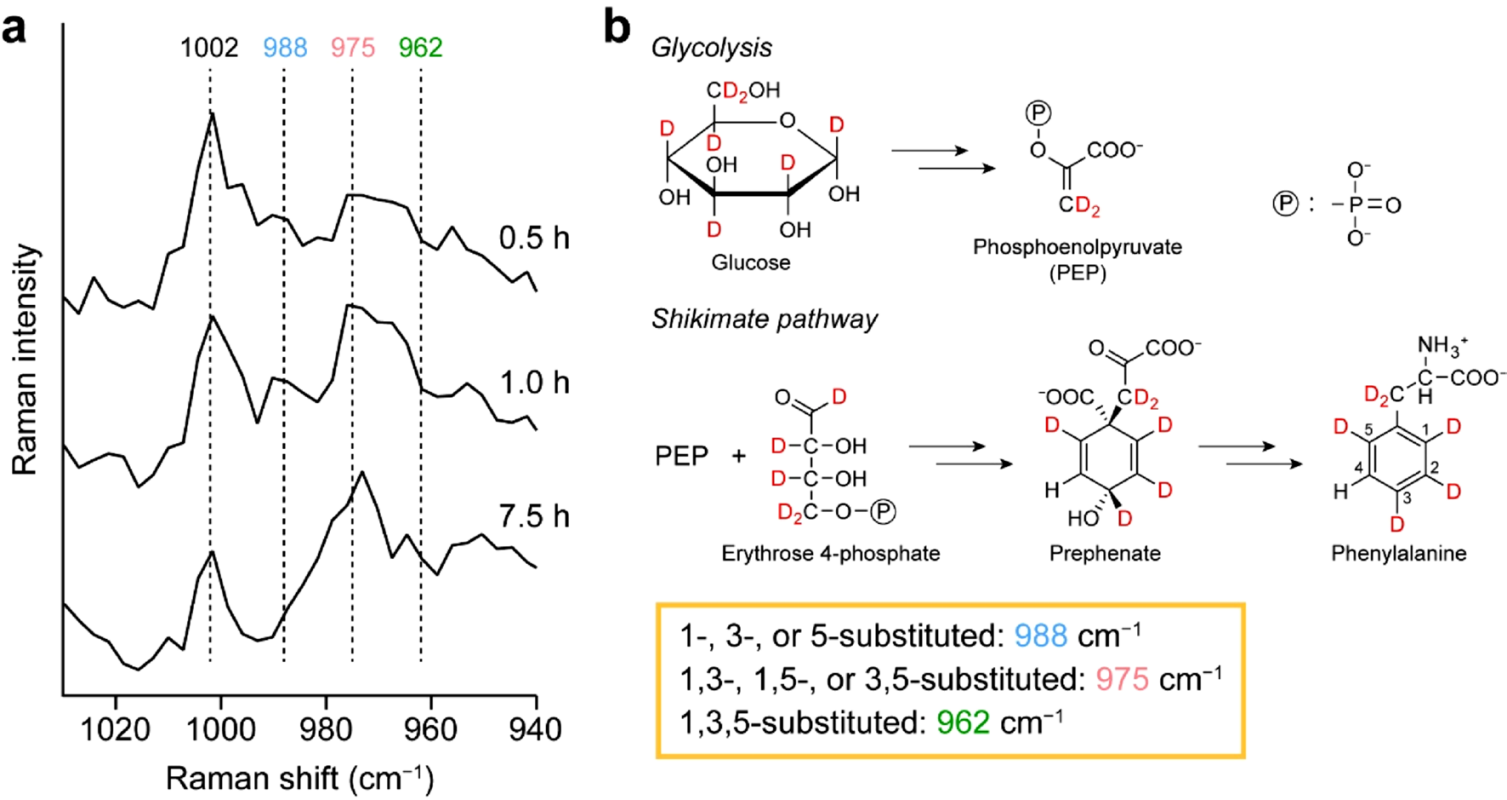
Biosynthetic incorporation of deuterium from D-labeled glucose into proteins. (**a**) Raman spectra in the 940–1030 cm^−1^ region of the *A. nidulans* hypha at 0.5, 1.0, and 7.5 h. Each spectrum is an average of the Raman spectra recorded at all positions inside the hypha and vertically offset for clarity of display. (**b**) Simplified scheme for the biosynthetic pathways of the aromatic amino acid phenylalanine from glucose precursor via glycolysis and the shikimate pathway. This scheme was drawn by using ChemDraw 15.1.0.144 software, https://www.perkinelmer.com/category/chemdraw.

Biosynthetic incorporation of deuterium into proteins can also be confirmed from the isotopic shift of the phenylalanine (Phe) ring-breathing band at 1002 cm^−1^ in the fingerprint region (Fig. 3a). At 0.5 h, only a single band is observed at 1002 cm^−1^, but at longer times (e.g., 1.0 and 7.5 h), another band comes out at ~ 975 cm^−1^. It has been known that this band is assigned to partially D-substituted Phe residues in proteins^15^. In microorganisms including fungi, the aromatic amino acid Phe is synthesized from glucose via glycolysis and the shikimate pathway (Fig. 3b). Tracing the flow of D label in the metabolic pathways, we see that deuteration can occur at 1-, 2-, 3-, and 5-positions on the phenyl ring, producing various isotopomers. Of these isotopomers, those in which any two of 1-, 3-, and 5-positions are substituted with deuterium show a Raman band at 975 cm^−1^ (Ref.^15^). Other isotopomers that show bands at 988 (1-, 3-, or 5-substituted) and 962 (1,3,5-substituted) cm^−1^ (Ref.^15^) were not observed in the present work (see Fig. 3a). One might consider that the 975 cm^−1^ band could then be used as a marker for D-labeled proteins rather than the deconvolved C–D stretching band at 2175 cm^−1^ described above. Unfortunately, however, this band is very weak and becomes visible only after averaging (recall that the spectra shown in Fig. 3a are an average of the spectra acquired at all points inside the hypha). Thus, we were unable to obtain reliable, high-contrast Raman images using the D-substituted Phe band at 975 cm^−1^ and decided to use the 2175 cm^−1^ band.

The remaining 2237 cm^−1^ component has a substantial contribution from extremely weak emission of reduced cytochrome *c*^36^, which is evident in the spectrum of *A. nidulans* grown in H medium (see the asterisk in Fig. 1b). It should therefore be insensitive to D labeling. We will provide further support for this assignment later based on the corresponding Raman images.

It might seem an oversimplification that we applied a Lorentzian deconvolution method to the C–D stretching manifold of glucose and proteins that would be a complex superposition of the CD, methylene (CD_2_), and methyl (CD_3_) modes. However, as studied in detail by Shi and coworkers^27^, very few CD_2_ and CD_3_ moieties are present in D-labeled proteins, and the C–D mode predominates, thereby justifying that a single Lorentzian band is effectively a good description of the C–D stretching band of D-labeled proteins. The actual C–D stretching band of intracellular D-labeled glucose could resemble that of glucose in aqueous solution possessing multiple peaks (Fig. 2a, dashed line). Thus, some contamination of the protein signal at 2175 cm^−1^ by the glucose signal might occur (see below).

### Time-lapse Raman images of D-labeled glucose and proteins

**Figure 4:**
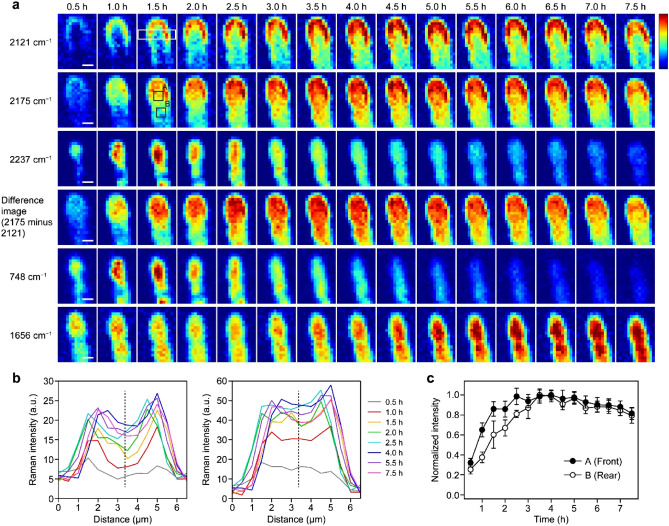
Time-lapse deuterium-labeled Raman imaging of the *A. nidulans* hyphal tip. (**a**) Time-lapse Raman images of the three deconvolved bands in the C–D stretching region at 2121, 2175, and 2237 cm^−1^, together with the difference images between the images at 2175 and 2121 cm^−1^ and Raman images of the 748 and 1656 cm^−1^ bands. In these images, Raman intensities are encoded in the “jet” color scale. The same color scale applies to Raman images in each row. White rectangular in the 2121 cm^−1^ image at 1.5 h indicates the region from which the line profiles shown in **b** were calculated; black squares (A and B) in the 2175 cm^−1^ image at 1.5 h indicate the regions from which the temporal profiles shown in **c** were obtained. Scale bar = 2 μm. (**b**) Line profiles of the 2121 and 2175 cm^−1^ images at 0.5, 1.0, 1.5, 2.0, 2.5, 4.0, 5.5, and 7.5 h. Dashed lines indicate the middle of the hypha. (**c**) Temporal profiles of the 2175 cm^−1^ band intensity in the front (A, filled circle) and rear (B, open circle) regions of the middle part of the hyphal tip. The intensity at each time point was calculated by averaging those at nine pixels within regions A and B and then normalized to the maximum value. Error bars represent standard deviation.

These Raman bands mainly contributed by D-labeled glucose and proteins allow for visualizing the spatiotemporal characteristics of glucose accumulation and protein metabolism in the hyphal tip. Figure 4a shows a time course of the Raman images for the three deconvolved bands at 2121, 2175, and 2237 cm^−1^, together with those constructed at the two fingerprint Raman bands at 748 and 1656 cm^−1^. The 1656 cm^−1^ band, which is called amide I (see Supplementary Table S1), exhibits no apparent D-shift and its intensity represents the total amount of proteins, whether unsubstituted or D-substituted.

The Raman image of the C–D stretching 2121 cm^−1^ band (D-labeled glucose) reveals a highly localized distribution pattern along the inner edge of the *A. nidulans* hyphal tip (Fig. 4a, first row). This characteristic inverted “U-shape” or horse-shoe shape, which reminds us of active endocytosis zone^30,38^ (see the Discussion section), is already appreciable in 1.0 h and becomes more and more prominent with time. Concurrently, the distribution of the 2121 cm^−1^ band extends to the rear part of the hyphal tip. This result suggests preferential accumulation along the edge, of D-labeled glucose taken up around the hyphal tip, and subsequent diffusion into the rest part.

In contrast, the C–D stretching 2175 cm^−1^ image displays high-intensity patterns (represented by red-to-yellow colors in the Raman images) not only along the hyphal edge but also in the central part of the apical region of the hypha (Fig. 4a, second row). The former may be attributable to the contribution of the overlapping 2121 cm^−1^ band, so we subtracted the 2121 cm^−1^ images from the 2175 cm^−1^ images. In the resulting difference images (Fig. 4a, fourth row), the inverted U-shaped pattern characteristic of D-labeled glucose is no longer evident and the intensities are distributed only in the middle of the hypha. To further see the difference in the distribution pattern between the 2121 and 2175 cm^−1^ images, we plot line profiles of these images along the *X*-axis (i.e., the direction to cross the hypha) by averaging three adjacent slices within the white-boxed area shown in the 2121 cm^−1^ image at 1.5 h. Consistent with the inverted U-shaped distribution pattern, the line profile of the 2121 cm^−1^ image (Fig. 4b, left) has a marked depression near the middle of the hypha particularly at earlier times (e.g., 1.0, 1.5, and 2.0 h), whereas that of the 2175 cm^−1^ image (Fig. 4b, right) is much flatter. The higher intensity of the 2175 cm^−1^ band (D-labeled proteins) in the central part of the apical region suggests that active protein synthesis occurs there by utilizing D-labeled glucose accumulated in the inverted U-shaped region.

A comparison of the dynamics provides additional insight into the protein metabolism observed here. To this end, we averaged the 2175 cm^−1^ band intensity at each culture time, at nine pixels inside the front (A) and rear (B) parts of the hyphal tip (designated by black squares in the 2175 cm^−1^ image at 1.5 h; see Fig. 4a) and plotted the averaged value against time (Fig. 4c). The front part A is located within 3–4 μm from the apex and the rear part B ~ 3 μm behind A. It is clear from Fig. 4c that the front part (filled circle) shows a faster rise than the rear part (open circle). To quantitatively estimate the intensity rise time, we attempted to simultaneously fit the two normalized temporal profiles $I\left(t\right)$, using the following phenomenological equation:

1 $$I\left(t\right)=1-\mathrm{exp}[-(t-{t}_{0})/\tau ]$$

where $\tau$ represents an exponential time constant and ${t}_{0}$ accounts for the uncertainty in time zero and/or certain lag time (Supplementary Fig. S1). $\tau$ was determined to be 0.76 (± 0.08) h for the front part and 1.4 (± 0.1) h for the rear part, so the rise is ~ 1.8 times faster in the front part than in the rear part. We note that the distinct spatial and temporal features revealed here are specific to newly synthesized D-labeled proteins and are different from those of the whole protein (see the amide I image at 1656 cm^−1^ shown at the bottom of Fig. 4a).

The Raman image at 2237 cm^−1^ (Fig. 4a, third row) shows remarkable resemblance to the cytochrome Raman image at 748 cm^−1^ (Fig. 4a, fifth row), in both spatial distribution and temporal evolution. This resemblance agrees well with our interpretation that the 2237 cm^−1^ band is attributable to cytochrome emission. Both 748 and 2237 cm^−1^ band intensities gradually diminish with time most likely because of the photobleaching of cytochromes caused by 532-nm laser irradiation^39^.

We repeated similar deuterium-labeled Raman imaging on several other hyphal tips than the one presented above and analyzed the data in exactly the same manner. The Raman images at 2121, 2175, and 2237 cm^−1^ obtained from one of those data are shown in Supplementary Fig. S2 as a typical example. The spatial patterns that we see in these Raman images are all similar to those in Fig. 4a, indicating the reproducibility of our results.

## Discussion

### Biological significance of the present findings

**Figure 5:**
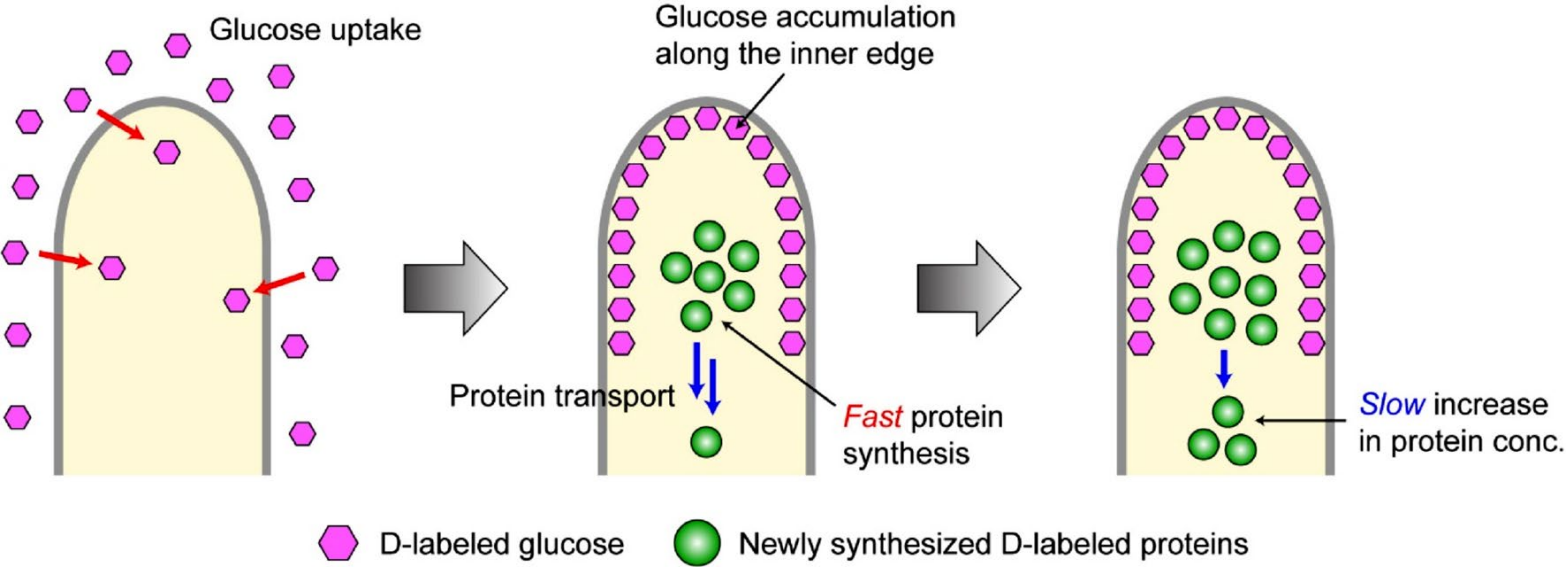
Cartoon picture showing the glucose uptake/accumulation and protein synthesis/transport processes in an *A. nidulans* hypha, as revealed by in vivo deuterium-labeled Raman imaging. conc. = concentration. This figure was created by using Adobe Illustrator CC 22.0.1 software, https://www.adobe.com/products/illustrator.html.

We have presented above time-lapse deuterium-labeled Raman imaging data on a single living *A. nidulans* hypha, from which the following scenario can be drawn about glucose and protein metabolism. First, uptake of extracellular D-labeled glucose occurs through glucose transporters (Fig. 5, left). Because glucose transporters have not been well studied in *A. nidulans*, its localization or active region is unknown^40–42^. Other transporters, such as purine transporters, are known to be located in the plasma membranes of posterior hyphae other than hyphal tips^43^. The predicted glucose transporter might have a unique function to sense glucose at growing hyphal tips. Another possibility is that glucose is taken up in other parts in addition to hyphal tips and transported to hyphal tips; however, such a mechanism is not known either.

Glucose taken into the hypha is preferentially accumulated along the inner edge of the hyphal tip (Fig. 5, middle), ultimately leading to the inverted U-shaped distribution palpable in the Raman image at 2121 cm^−1^ (Fig. 4a, first row). As soon as glucose is accumulated (in 1–2 h), deuterium of D-labeled glucose is anabolically incorporated into proteins near the center of the hyphal apical region (Fig. 5, middle). This localization pattern is reminiscent of a “dynein loading zone” or “sorting endosomes” known in filamentous fungi^44,45^. Endocytosis is active around hyphal tips except for the apex, which is occupied with exocytosis^30,38^. Endocytic vesicles fuse to early endosomes, which move bidirectionally along microtubules^46^. In particular, those around hyphal tips show this behavior at the central part of hyphal tips called the dynein loading zone and/or sorting endosomes^44,47^. Notably, early endosomes carry RNA-binding proteins at cytoplasmic side and transport mRNAs over long distances in hyphae^48^. Moreover, early endosomes transport polysomes as well, leading to local protein translation^49^. The new protein synthesis revealed in this study might occur at the dynein loading zone and/or sorting endosomes. The slower increase (by a factor of ~ 1.8) in protein concentration in the rear part of the hyphal tip very likely results from diffusion and/or transport of the proteins newly synthesized in the front part (Fig. 5, right). The cartoon picture in Fig. 5 illustrates the putative dynamics of glucose accumulation and protein metabolism/transport, which has been unraveled for the first time by in vivo deuterium-labeled Raman imaging.

### Possible effect of laser irradiation

A critical issue that needs to be addressed is that the hypha imaged in the present study was not growing. This phenomenon is presumably due to laser irradiation during imaging. We carefully chose minimum required laser power (4.4 mW) and exposure time (1 s) in the experiment, but the effects of constant laser irradiation on hyphal tip growth still seem unavoidable. The observed dynamics could then reflect some hyphal response to laser-induced cellular damage rather than metabolism. However, we stress that the characteristic inverted U-shaped distribution of D-labeled glucose is clearly visible at 0.5 h, the very beginning of the imaging experiment. Another hypha we measured (Supplementary Fig. S2) also showed glucose accumulation at the earliest imaging time and afterwards grew slightly. In addition, we observed the emergence of the Raman band of D-substituted Phe (Fig. 3a), which indicates without doubt protein metabolism. Taken together, although laser irradiation could affect hyphal tip growth, our main findings of the metabolic dynamics are not affected.

### Comparison with other approaches

Prior studies also used Raman microspectroscopy coupled with deuterium labeling to elucidate microbial metabolism at the single-cell level^15,18^. The spatial resolution of confocal Raman microspectroscopy, which is dictated by the diffraction limit of the light used, is typically a few hundred nanometers and insufficient to resolve well the inner structure of individual bacterial cells, such as *Escherichia coli* and *Pseudomonas putida*. Thus, Raman imaging was not performed in those studies. The work on *S. pombe* by Noothalapati and Shigeto^19^ is one of few examples that carried out single-cell imaging of microorganisms by taking advantage of the larger cell size of yeasts. The present work is its natural extension but provides more detailed, quantitative insight into fungal metabolism.

Even larger animal cells and tissues were recently studied using SRS with the deuterium labeling technique^26,27^. SRS is much superior to spontaneous Raman spectroscopy in imaging speed, but so far it is unable to obtain the entire Raman spectrum due to the detection of Raman signals only at several limited wavenumbers (i.e., those within the C–D stretching band). In contrast, as shown in Fig. 1b, we are able to access almost full vibrational information at once covering both the fingerprint region (500–1800 cm^−1^) and the C–H/C–D stretching region. There are a number of vibrational modes in the fingerprint region available as a marker for specific metabolites: the amide I and Phe ring-breathing modes for proteins, C = C double-bond stretching for lipids^19,23^, to name just a few. Although the resonance Raman bands of cytochromes observed in this study do not exhibit appreciable D-shift, they are predicted to do so upon ^13^C labeling. We thus argue that our method using spontaneous Raman spectroscopy outperforms the state-of-the-art SRS imaging in terms of molecular specificity. This advantage cannot be overemphasized, because analysis of as many Raman bands as possible of different molecules enables us to probe different metabolisms simultaneously. The high molecular specificity of our method will also be beneficial when it is applied to the imaging study of microbial symbiosis (e.g., fungal–bacterial interactions^50^), which is in progress in our laboratory.

A molecular-level understanding of cellular metabolism toward elucidation of polarized growth in filamentous fungi will have important implications for many fields of research and development, such as efficient production of useful secondary metabolites^51,52^. Metabolic imaging using stable isotope-labeled Raman spectroscopy offers a facile, nondestructive tool with subcellular spatial resolution for studying how spatial variations in different (but interconnected) metabolic activities lead to the sustained growth and homeostasis of fungal hyphae. By using a multiplex nonlinear Raman technique^53^, we can alleviate the drawback of spontaneous Raman microspectroscopy in imaging speed and make it fully applicable to tracking of faster metabolic dynamics in a wider spatial range.

## Methods

### Reagents, fungal strain, and culture conditions

All reagents used were purchased from FUJIFILM Wako Pure Chemical unless otherwise noted. *Aspergillus nidulans* strain TN02A3 (Ref.^54^) was used in this study. Minimal medium (pH 6.5) for culturing *A. nidulans* was prepared according to the literature^55^, which contained NaNO_3_ (6.0 g L^−1^), KCl (0.52 g L^−1^), MgSO_4_·7H_2_O (0.52 g L^−1^), KH_2_PO_4_ (1.52 g L^−1^), and Hutner’s trace elements^56^ (1.0 mL L^−1^), plus pyridoxine–HCl (0.2 mg L^−1^), uridine (1.2 g L^−1^), and uracil (1.12 g L^−1^) as supplements^57^. Either unlabeled glucose or partially deuterated (D-labeled) glucose (1,2,3,4,5,6,6-*d*_7_, 97 atom % D; Sigma-Aldrich) was added at 10 g L^−1^ as the primary carbon source.

*A. nidulans* was grown at room temperature for several days on an agar plate, which was H medium solidified by adding 1.5% agar (nacalai tesque). Spores were picked from the plate, inoculated into H medium in a glass Petri dish, and cultured at room temperature for 24–36 h. Subsequently, H medium was replaced with D medium. Raman imaging measurements on a single living hypha of *A. nidulans* were performed every 30 min up to 7.5 h after replacement of H medium with D medium (defined as 0 h).

### Raman imaging

Time-lapse Raman imaging was performed with an upright, 532-nm-excited confocal Raman microspectrometer (XploRA Nano, Horiba), as in our previous study^31^. A 6.5 × 9.5 μm^2^ area involving a hyphal tip of *A. nidulans* that attached to the bottom of the Petri dish was raster-scanned at 0.5 μm intervals and the Raman spectrum at each point was acquired with a 1 s exposure time and 4.4 mW laser power. The laser beam was focused with a 60 × , NA = 1.0, water-immersion objective (LUMPLFLN60XW, Olympus). Backscattered light was collected with the same objective and analyzed with a spectrograph. A 1200 mm^−1^ grating was used so as to cover a wide spectral window from ~ 490 to ~ 3085 cm^−1^ with a 5 cm^−1^ spectral resolution. The same area was imaged at 0.5 h intervals from 0.5 h to 7.5 h after H medium was replaced with D medium, yielding 15 sets of Raman imaging data, each of which consists of 280 (= 14 × 20) spectra having 1024 pixels. The spatial resolution of the Raman microspectrometer was estimated to be 0.57 μm in the lateral (*XY*) direction and 7.4 μm in the axial (*Z*) direction. The reference Raman spectrum of D-labeled glucose dissolved in water (100 g L^−1^) was obtained by averaging 25 spectra recorded with a 1 s exposure time.

### Spectral analysis

Raman imaging data were subjected to the following pretreatment. Spiky artifacts due to cosmic rays were manually removed^31^. Next, because cellular Raman spectra obtained with an exposure time as short as 1 s typically entail high noise levels, singular value decomposition (SVD)-based denoising^58,59^ was performed. The 15 Raman imaging datasets were combined to form a 1024 × 4200 matrix, and SVD of the combined data was computed. Nine singular components associated with large singular values and meaningful singular vectors were retained to reconstruct noise-reduced imaging data (see Supplementary Figs. S3 and S4 for details). Finally, the background signal due to D medium (and possibly the glass substrate) was subtracted from the denoised spectra using a method proposed by Sugawara et al.^60^.

The pretreated Raman spectra were used to generate intensity distribution maps (Raman images) for Raman bands of interest. Least-squares curve fitting using three Lorentzian functions and a linear baseline was performed to deconvolve the multipeak C–D stretching band and to obtain the area intensities of the underlying subbands. For other isolated Raman bands, a simpler protocol was adopted in which the area between the band contour and a straight line connecting the edges of the band was found by numerical integration^59^. All spectral analysis was performed in Igor Pro 8 (WaveMetrics).

## Supplementary information

Supplementary Information.
