# Supplementary material for: Deuterium-labeled Raman tracking of glucose accumulation and protein metabolic dynamics in Aspergillus nidulans hyphal tips
Source: Sci Rep. 2021 Jan 14;11:1279. doi: 10.1038/s41598-020-80270-9 (PMC7809412; doi:10.1038/s41598-020-80270-9)
Supplement: Supplementary file 1 — Supplementary Information. [file 41598_2020_80270_MOESM1_ESM.pdf]

Supplementary Information

Deuterium-labeled Raman tracking of glucose accumulation and protein metabolic dynamics in *Aspergillus nidulans* hyphal tips

Mitsuru Yasuda[1†], Norio Takeshita[2] & Shinsuke Shigeto[1*]

[1]Department of Chemistry, School of Science and Technology, Kwansei Gakuin University, Sanda, Hyogo 669-1337, Japan.
[2]Microbiology Research Center for Sustainability (MiCS), Faculty of Life and Environmental Sciences, University of Tsukuba, Ibaraki, Tsukuba 305-8572, Japan.

†Present address: Department of Pharmacology, School of Medicine, Keio University, Tokyo 160-8582, Japan
*Corresponding author. E-mail: shigeto@kwansei.ac.jp

Table of contents

Supplementary Figure S1 .... S2
Supplementary Figure S2 .... S3
Supplementary Figure S3 .... S4
Supplementary Figure S4 .... S5
Supplementary Table S1 .... S6
Supplementary Table S2 .... S6

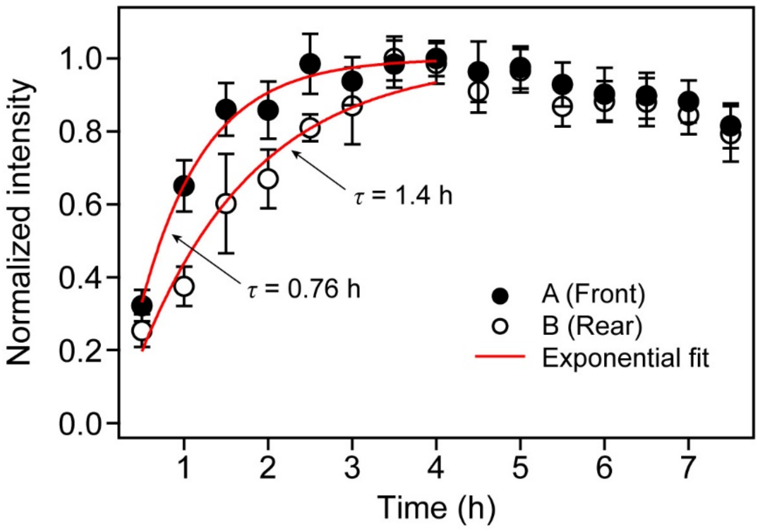

Supplementary Figure S1. Fitting analysis of the normalized temporal profiles of the 2175 cm^{-1} band intensity in the front (A, filled circle) and rear (B, open circle) regions of the middle part of the hyphal tip. The rise part (0.5–4.0 h) of both data were simultaneously fit to eq 1 with a common value of t_0. The best fits are shown with red lines. The τ value was obtained to be 0.76 (±0.08) h for the front part and 1.4 (±0.1) h for the rear part, and the t_0 value to be 0.19 (±0.06) h. Note that eq 1 was used solely for better approximation of the temporal profiles and was not based on any physical/biological model.

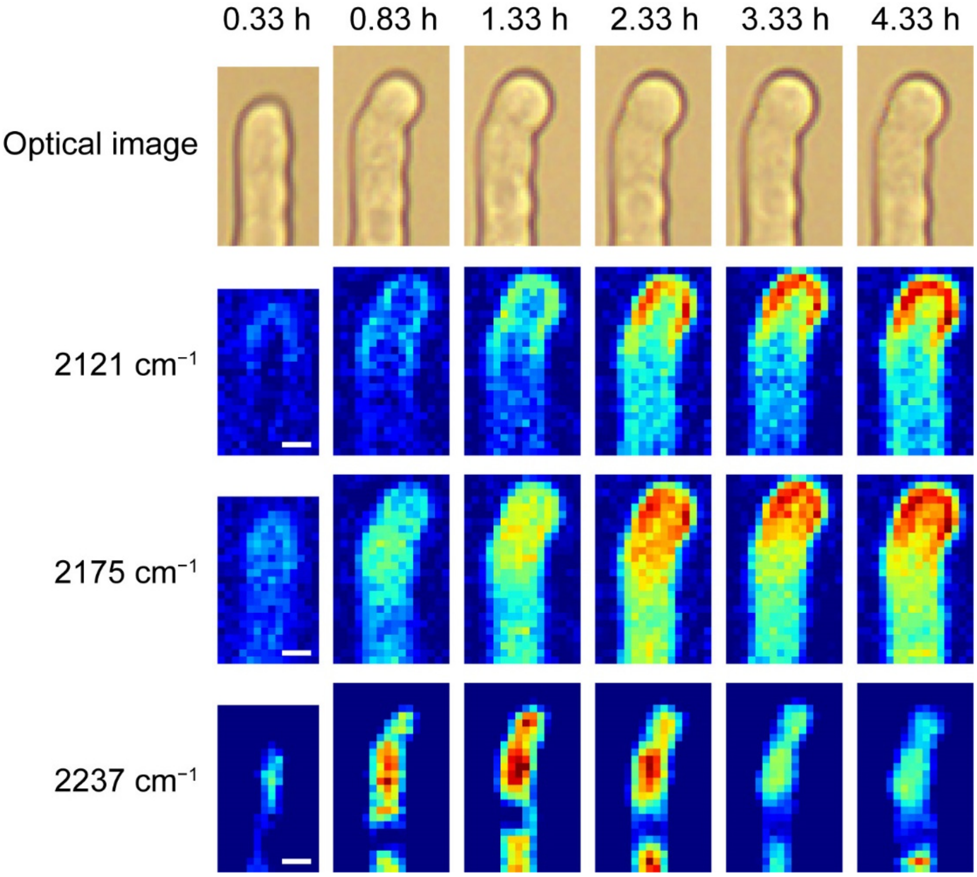

Supplementary Figure S2. Time-lapse deuterium-labeled Raman imaging of another *A. nidulans* hyphal tip. From top to bottom: Optical images and Raman images of the three deconvolved bands in the C–D stretching region at 2121, 2175, and 2237 cm^{-1}. Band deconvolution analysis was performed in the same manner as for Fig. 4. Scale bar = 2 µm.

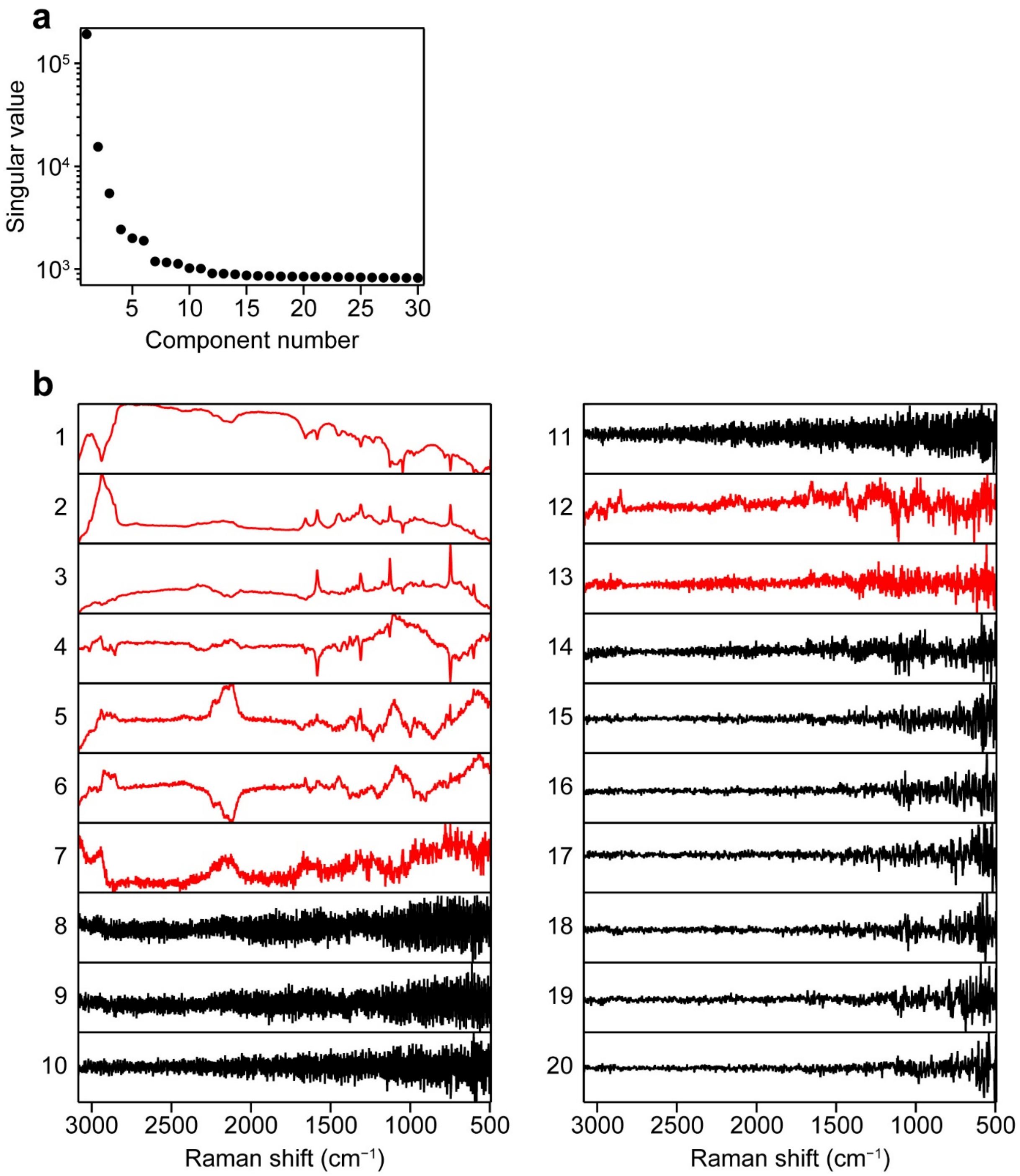

Supplementary Figure S3. Noise reduction using singular value decomposition (SVD). (**a**) Logarithmic plot of the largest 30 singular values. (**b**) Singular vectors of the largest 20 singular components. All spectra are shown in different scales. Red color highlights the nine components (1–7, 12, and 13) that were retained to reconstruct denoised data. They have large singular values and exhibit meaningful spectral patterns. Although components 8–11 have greater singular values than components 12 and 13 do, their singular vectors appear to be dominated by noises without sharp Raman features. Hence, they were not included in the reconstruction. See Supplementary Fig. S4 for justification of this procedure.

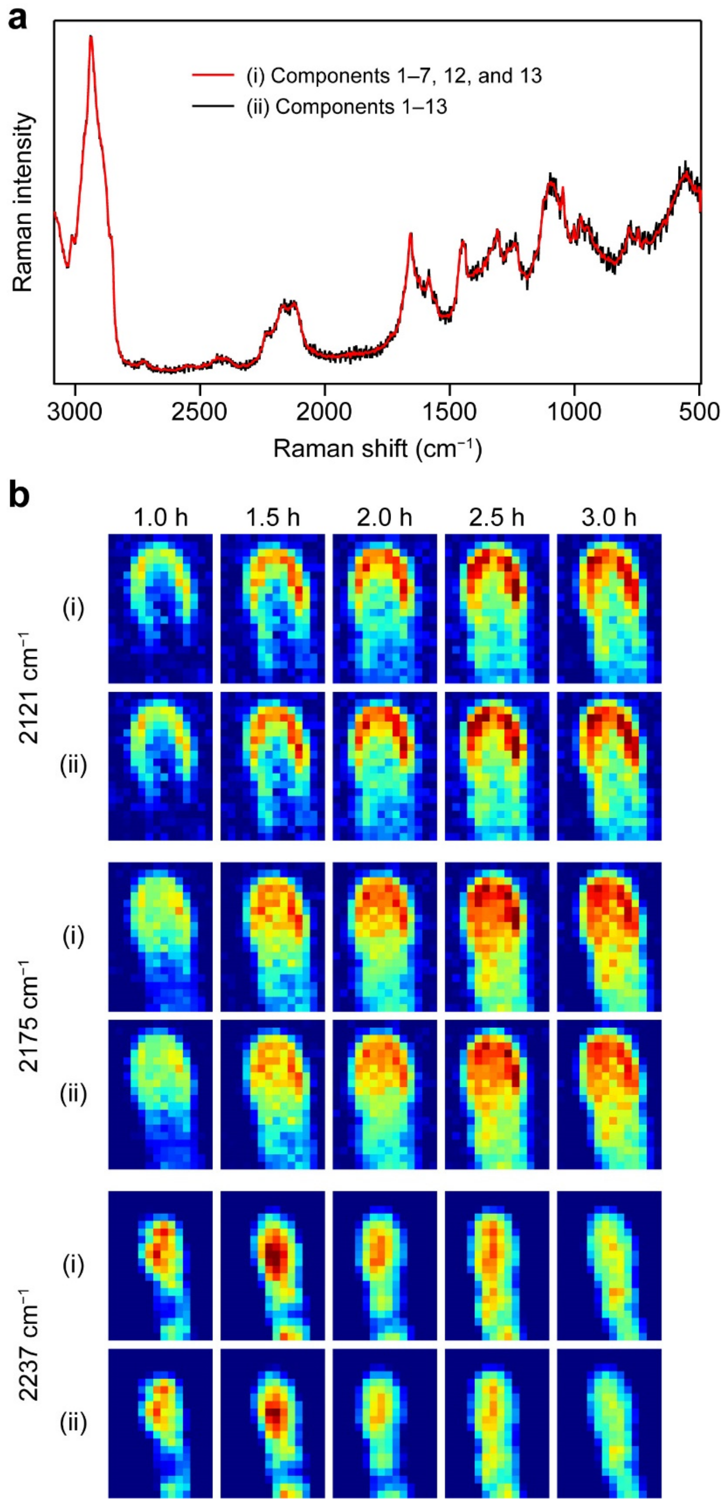

Supplementary Figure S4. Noise reduction by SVD using different numbers of singular components. (**a**) Comparison of a representative space-resolved Raman spectrum subjected to SVD-based denoising in which (i) 9 singular components 1–7, 12, and 13 (red line) and (ii) 13 singular components 1–13 (black line). (**b**) Comparison of the Raman images of the 2121 (top), 2175 (middle), and 2237 (bottom) cm^{-1} bands at selected time points, obtained from the SVD-treated data with (i) 9 components (same images as in Fig. 4a) and (ii) 13 components retained.

Supplementary Table S1. Assignment of major Raman bands observed in the Raman Spectrum of *Aspergillus nidulans* hyphal tip grown in H medium.

Raman shift (cm^{-1})	Assignment	Major molecular components
2940	C–H str.	Proteins/lipids
1656	Amide I	Proteins
1583	ν_{19}, $C_{\alpha}C_{m}$ asym. str.	Cytochromes
1456	CH_3 deg. deform.	Proteins
1440	CH_2 scissor.	Proteins/lipids
1337	CH bend.	Proteins
1313	ν_{21}, $C_{m}H$ bend.	Cytochromes
1242	Amide III	Proteins
1128	ν_{14}, $C_{\beta}C_{1}$ sym. str.	Cytochromes
1003	Phenylalanine ring breath.	Proteins
853	Tyrosine doublet	Proteins
748	ν_{15}, pyrrole breath.	Cytochromes
601	ν_{24}, pyrrole asym. deform.	Cytochromes

Abbreviations: str., stretching; deform., deformation; scissor., scissoring; bend., bending; breath., breathing; sym., symmetric; asym., asymmetric; deg., degenerate.

Supplementary Table S2. Peak positions and band widths of the three Lorentzian subbands used to fit the C–D stretching band.

Peak position (cm^{-1})	Band width[a] (cm^{-1})
2121	62
2175	75
2237	35

[a]Full width at half-maximum.
